# Knee position affects medial gastrocnemius and soleus activation during dynamic plantarflexion: no evidence for an inter-muscle compensation in healthy young adults

**DOI:** 10.1242/bio.061810

**Published:** 2024-12-30

**Authors:** Bálint Kovács, Dániel Csala, Song Yang, József Tihanyi, Yaodong Gu, Tibor Hortobágyi

**Affiliations:** ^1^ Faculty of Sport Science, Ningbo University, Ningbo 310211, China; ^2^ Department of Kinesiology, Hungarian University of Sports Sciences, Alkotás utca 44-48, Budapest 1123, Hungary; ^3^ Department of Biomedical Engineering, Faculty of Engineering, The Hong Kong Polytechnic University, Hong Kong TU428, China; ^4^ Department of Sport Biology, Institute of Sport Sciences and Physical Education, University of Pécs, Pécs 1123, Hungary; ^5^ Department of Neurology, Somogy County Kaposi Mór Teaching Hospital, Kaposvár 7400, Hungary; ^6^ Center for Human Movement Sciences, University of Groningen Medical Center, Groningen 9700, Netherlands

**Keywords:** HD-EMG, Dynamometric, Plantarflexors

## Abstract

Knee joint position influences ankle torque, but it is unclear whether the soleus compensates to counteract the reductions in gastrocnemius output during knee-flexed versus knee-extended plantarflexions. Therefore, the purpose of this study was to determine the effects of knee joint position and plantarflexion contraction velocity on ankle plantarflexion torque and electromyography activity of the medial gastrocnemius and soleus in healthy young adults. Healthy male participants (*n*=30) performed concentric plantar flexions in a custom-built dynamometer from 15° dorsiflexion to 30° plantarflexion at gradually increasing velocities during each contraction at 30, 60, 120, 180, and 210° s^−1^ in a supine position with the knee fully extended and while kneeling with the knee fixed in 90° flexion. Two 16-channel linear electromyographic (EMG) arrays were placed over the medial gastrocnemius and soleus muscles. Plantarflexion torque during flexed-knee versus extended-knee plantarflexions was 31% lower (*P*=0.002) averaged across the five contraction velocities. The overall EMG activity of the medial gastrocnemius was 35% lower (*P*=0.002) during knee-flexed versus knee-extended plantarflexions. In the first half of plantarflexions at slower contractions, soleus EMG activity was 15% and 28% higher (both *P*=0.002) in knee-flexed versus knee-extended plantarflexion, respectively. We conclude that knee position affects medial gastrocnemius and soleus activation during dynamic plantarflexion, with plantarflexion torque being smaller in the knee-flexed versus knee-extended position. However, we found no evidence that changes in soleus activation would compensate for the decrease in medial gastrocnemius activation.

## Teaser

**Summary:** Knee position impacts plantarflexor muscle activation and reduces plantarflexion strength, with decreased medial gastrocnemius activity but no compensatory increase in soleus activation.

## INTRODUCTION

The triceps surae muscle is a key determinant of plantarflexion (PF) torque during daily activities such as walking and running (Avancini et al., 2015; Cresswell et al., 1995; Dorn et al., 2012; Lai et al., 2018; Schache et al., 2014). Triceps surae consists of the medial gastrocnemius (MG), the lateral gastrocnemius and the soleus (SOL) muscles. These muscles are structurally diverse (Fukunaga et al., 1992; Kovács et al., 2020), and their relative contribution to the ankle PF torque differs (Avancini et al., 2015). SOL and MG muscles contribute differently to ankle PF torque due to distinct anatomical and physiological characteristics. The SOL compared with MG has a significantly larger physiological cross-sectional area (Albracht et al., 2008; Fukunaga et al., 1992) that allows it to have a higher relative contributions to PF torque (Cresswell et al., 1995). In addition, the SOL and MG, on average, consist of ∼80% versus 60% slow-twitch fibers, respectively (Gollnick et al., 1974; Ohira et al., 1999; Tirrell et al., 2012). Beside the architectural differences, SOL has a highly compartmentalized structure (Bolsterlee et al., 2018), which may lead to varying functional interpretations based on electromyographic (EMG) electrode placement. In addition, the two heads of the gastrocnemius are biarticular, crossing the ankle and knee joints to assist in both ankle PF and knee flexion. Such structural and functional differences between the SOL and MG allow for complementary roles in PF. Indeed, the position of the ankle and knee joints during PF strongly affects the force-producing capacity of each gastrocnemius. In particular, the position of the knee joint during PF seems to affect the relative contribution of MG to PF torque (Avancini et al., 2015; Cronin et al., 2011, 2013). On the other hand, SOL is a monoarticular muscle, and the position of the knee joint during PF does not seem to affect its contribution to PF torque (Arampatzis et al., 2006). Aligned with this assumption, SOL fascicle length remains unchanged during weak isometric contractions when the knee is passively flexed (Kawakami et al., 1998; Lauber et al., 2014). In contrast, initial MG fascicle length is shorter when the is knee flexed versus when the knee is extended (Avancini et al., 2015). In the knee-flexed condition during PF, SOL activity increases possibly in compensation for the force deficit caused by reductions in neural activation and fascicle length of the MG to maintain a constant PF torque (Lauber et al., 2014).

Maximal voluntary isometric PF torque is significantly lower when the knee is in a flexed versus extended position (Avancini et al., 2015; Kunugi et al., 2022; Shinohara et al., 2006). The reduction in ankle torque during knee-flexed PF is presumably related to the operation of the gastrocnemius muscle fascicles on the ascending limb of the force-length curve, decreasing the force-generating capacity of these fascicles (Hahn et al., 2011). Consequently, gastrocnemius EMG activity decreases as the knee is flexed during isometric PF (Arampatzis et al., 2006; Avancini et al., 2015; Hahn et al., 2011; Kunugi et al., 2022; Shinohara et al., 2006) because gastrocnemius fascicles are at a sub-optimal, shorter length, which can increase the inhibition of the MG motoneuron pool, reducing PF torque (Lauber et al., 2014). It is conceivable that neuromuscular compensation mechanisms are invoked to counteract reductions in PF torque generated with the knee flexed versus the knee extended. Indeed, the activation distribution of the MG muscle changes because distal MG regions actually become more, instead of less, activated as the knee is flexed during isometric ankle PF (Avancini et al., 2015). In addition, SOL activation might also increase (Arampatzis et al., 2006; Lauber et al., 2014; Shinohara et al., 2006), but this is not a universal finding because SOL activation can remain unchanged during knee-flexed versus knee-extended isometric PF (Farrag et al., 2022; Hali et al., 2019; Shinohara et al., 2006). Nevertheless, an increase in SOL activation in compensation for a decrease in MG activation is possible (Lauber et al., 2014; Tamaki et al., 1996).

Even less is known about the effects of knee joint position on MG-SOL activation during dynamic conditions (Hirata et al., 2016; Liu et al., 2020). One reason is that, during dynamic conditions, including isokinetic PFs, bipolar electrodes can record activation from a limited pickup volume of muscle. Such recordings might not frankly represent the inhomogeneous distribution of activity within the muscle and provide a biased estimate of muscle activity. To our knowledge, the effects of knee position on MG and SOL regional activation during dynamic PF have not yet been examined with high-density electromyography (HD-EMG) (Enoka, 2019). Therefore, the purpose of this study was to determine the effects of knee joint position and PF contraction velocity on ankle PF torque and activation amplitude and distribution of the MG and SOL in healthy young adults. While the difference in movement between skin and muscle is a limitation of HD-EMG during dynamic muscle contractions, the greater pick-up area of HD-EMG versus the bipolar surface electrode montage could characterize muscle activity in more detail.

The force-length and force-velocity properties of a muscle also affect muscle forces needed to generate ankle torque (Bohm et al., 2019). At a given operational fiber length, a lower versus higher contraction velocity allows a muscle to generate greater muscle force (Hill 1938). Therefore, increased angular rotation of the ankle (i.e. faster contraction velocity) will reduce muscle force output and, subsequently, neural activation of the muscle according to the force-velocity relationship (Hill 1938). These dynamics may impact the EMG activity pattern of the SOL differently than MG, because SOL primarily consists of slow-twitch fibers that exhibit lower rates of time-dependent cross-bridges (Fletcher and MacIntosh, 2017). Consequently, higher contraction velocities could lower the force-generating capacity of SOL, resulting in reduced EMG activity. Therefore, variation in knee joint position is likely to pose a dissimilar effect on MG and SOL EMG activity at different contraction velocities (Farrag et al., 2022), but these effects have not yet been fully characterized with respect to the position of the knee during PF. We expect that contraction velocity differentially affects MG and SOL activation, and subsequently torque generation, during PF in a knee-flexed position compared to a knee-extended position. Such differences would reflect these muscles' adaptive capacities to optimize force production during dynamic activities such as walking and running. During such activities, the position of the ankle joint and contraction velocity of muscles surrounding it both vary. Based on these data we expect to find that: (1) during flexed-knee PF, EMG activity in the SOL muscle will increase along its entire length to compensate for the reduction in MG activity; (2) this compensatory mechanism may become less pronounced as PF contraction velocity increases; and (3) we also expect lower PF torques when PF is performed in a knee-flexed versus knee-extended position independent of contraction velocity. Additionally, using a gradually increasing versus constant angular velocity during PF may approximate natural muscle contractions more closely, as real-life movements typically involve non-constant joint velocities.

## RESULTS

### Torque data

**Fig. 1.**
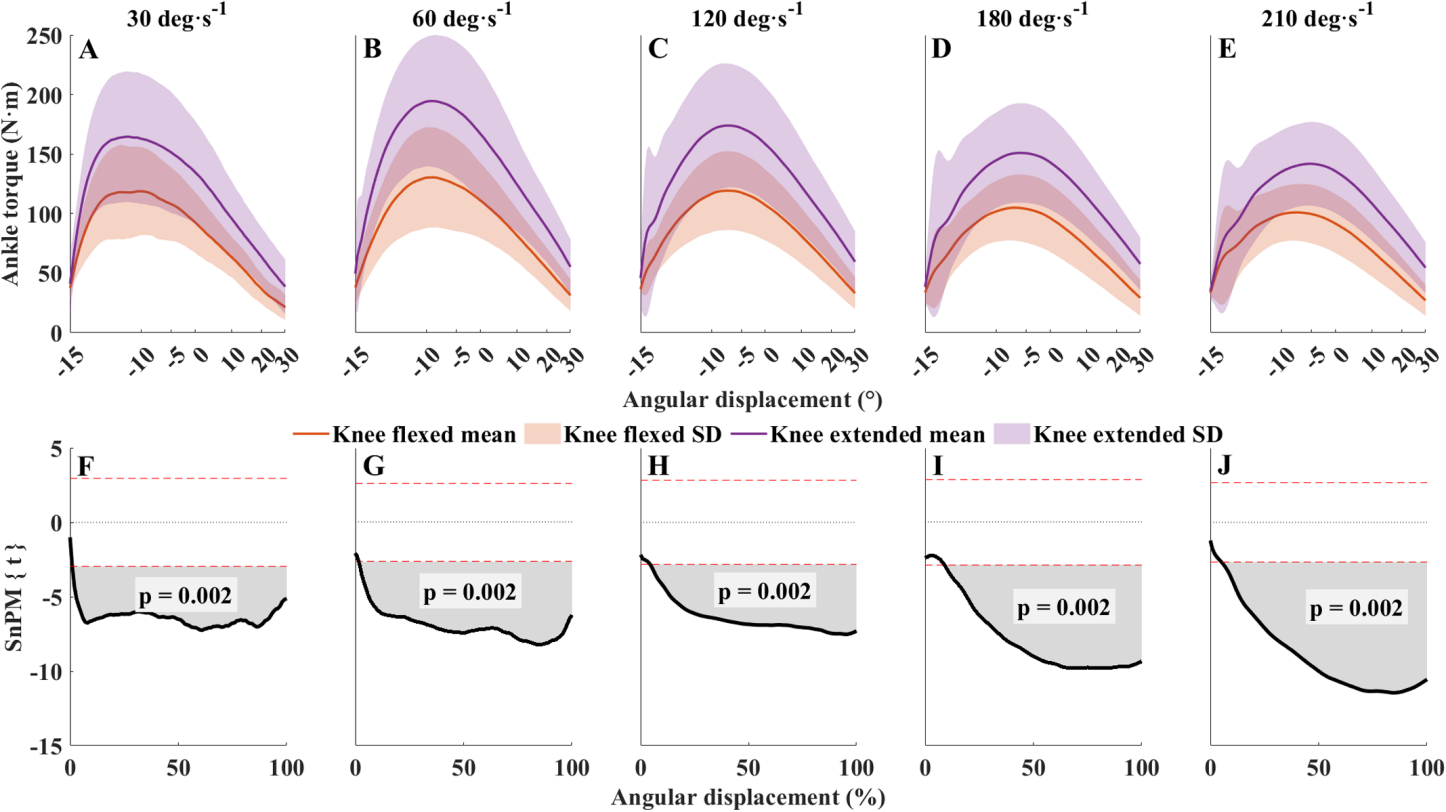
**Effects of knee position on plantarflexion torque.** (A-E) Mean ankle joint position-torque curves at 30, 60, 120, 180 and 210° s^−1^ (*n*=30). (F-J) Corresponding comparisons between knee-flexed and knee-extended joint torque values. Panels F-J: SnPM{t} statistics for paired *t*-tests (solid black lines) and mean difference. Dashed horizontal red lines are critical thresholds (t∗) calculated for α significance level defining supra-threshold clusters for SnPM{t} trajectories. *P*-values were calculated for each supra-threshold cluster. Spacing between the *x*-axis markers in A-E are gradually decreasing toward the end of the contraction, representing the continuously accelerating contraction velocity. In F-J, the *x*-axis was set to the normalized contraction time (0-101 points). Colored bands of shading denote ±1 s.d. Orange, knee flexed; purple, knee extended.

Knee-flexed versus knee-extended peak PF torque was lower at each contraction velocity (Fig. 1). Additionally, peak torque and mechanical work decreased proportionately at higher angular velocities in both knee joint positions. When PF was performed with the knee flexed, ankle torque was lower (*P*=0.002) during the contraction (from ∼2% to 100%) except at the beginning of the PF (from 0 to ∼2%) at all velocities (Fig. 1). The highest peak torque occurred at 30° s^−1^ with the knee extended (178.7±56.2 N·m). The lowest peak torque occurred at 210° s^−1^ with the knee flexed (90.1±21.5 N·m). The difference in peak ankle torque was the highest at 60° s^−1^ (112.57±37.37 N·m versus 167.73±48.90 N·m (i.e. a difference of 55.1 N·m or 33%); the smallest difference was at 210° s^−1^ (34.9 N·m - 28%).

### EMG data

**Fig. 2.**
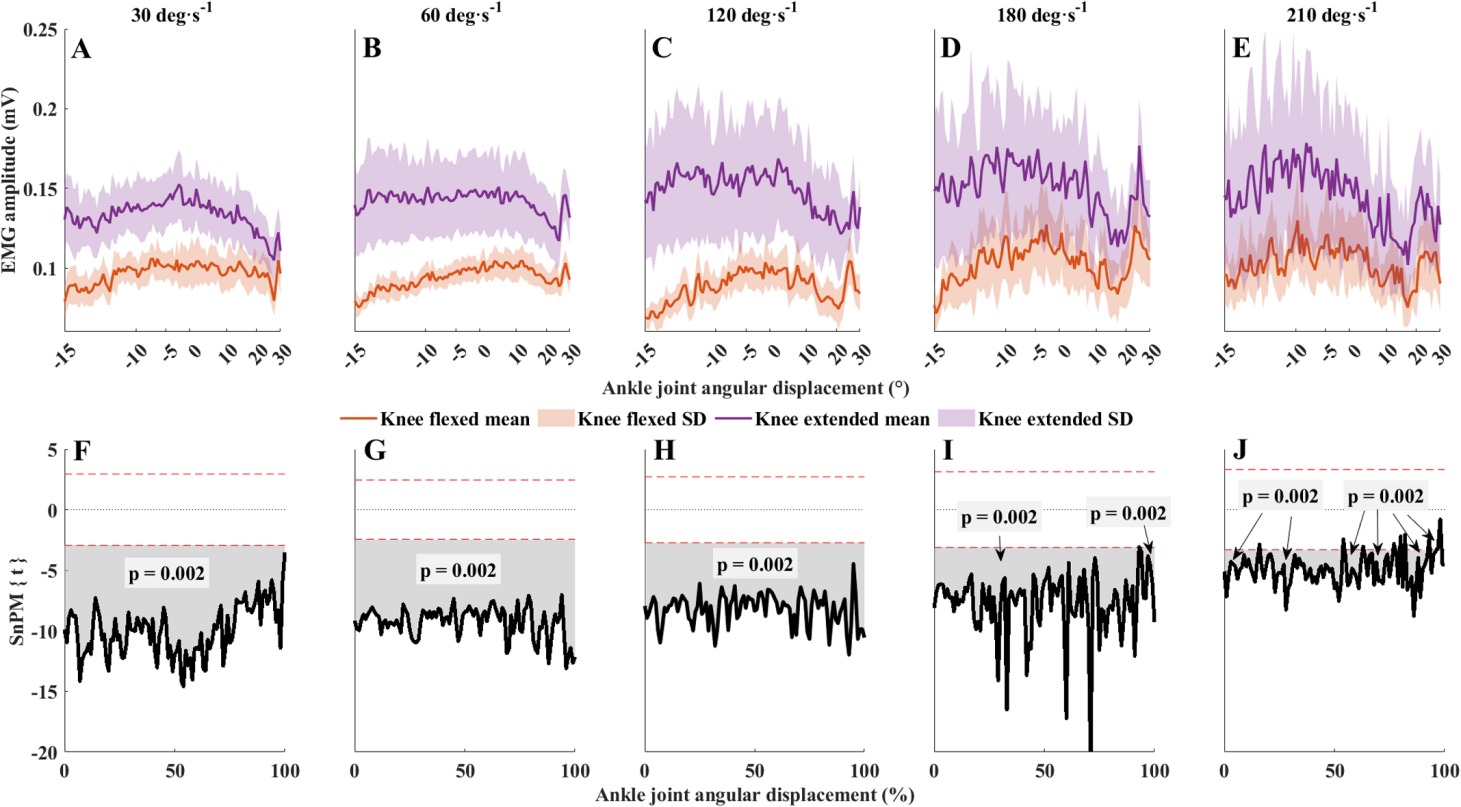
**Overall EMG activity (mean±s.d.) of the MG.** (A-J) Contraction velocities (A-E) and the corresponding comparisons between knee-flexed and knee-extended EMG values (F-J) are presented. Panels F-J: SnPM{t} statistics for paired *t*-tests (solid black lines) and mean difference. Dashed horizontal red lines are critical thresholds (t∗) calculated for α significance level defining supra-threshold clusters for SnPM{t} trajectories. *P*-values were calculated for each supra-threshold cluster. Spacing between the *x*-axis markers in A-E are gradually decreasing toward the end of the contraction, representing the continuously accelerating contraction velocity. In F-J, the *x*-axis was set to the normalized contraction time (0-101 points). Colored bands of shading denote ±1 s.d. Orange, knee flexed; purple, knee extended.

**Fig. 3.**
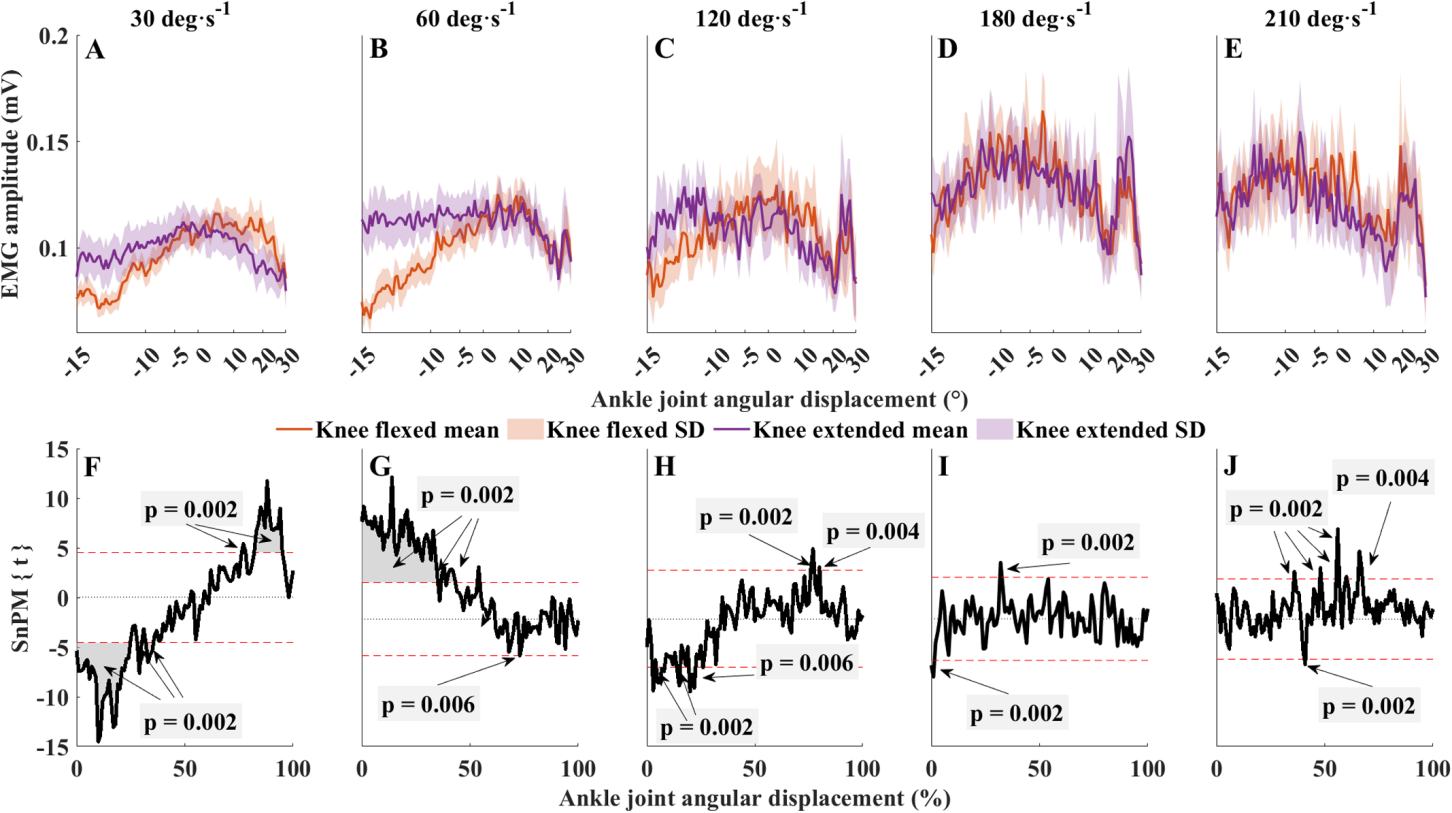
**Overall EMG activity (mean±s.d.) of the SOL.** (A-J) Contraction velocities (A-E) and the corresponding comparisons between knee-flexed and knee-extended EMG values (F-J) are presented. Panels F-J: SnPM{t} statistics for paired *t*-tests (solid black lines) and mean difference. Dashed horizontal red lines are critical thresholds (t∗) calculated for α significance level defining supra-threshold clusters for SnPM{t} trajectories. *P*-values were calculated for each supra-threshold cluster. Spacing between the *x*-axis markers in A-E are gradually decreasing toward the end of the contraction, representing the continuously accelerating contraction velocity. In F-J, the *x*-axis was set to the normalized contraction time (0-101 points). Colored bands of shading denote ±1 s.d. Orange, knee flexed; purple, knee extended.

Differences between knee-flexed and knee-extended EMG activity of MG and SOL are shown in Figs 2 and 3, separately for each contraction velocity. A knee joint position-MG EMG interaction was detected in a large period throughout the angular displacement of 4-100% (*F*>20, *P*=0.002; Fig. S1). When the overall EMG activity was averaged, the mean MG amplitudes in knee-flexed PF were lower by 26% at 30° s^−1^, 62% at 60° s^−1^, 51% at 120° s^−1^, 21% at 180° s^−1^ and 13% at 210° s^−1^ l compared to knee-extended PF. Post hoc analyses showed that the magnitude of reduction in EMG activity of MG in knee-flexed PF was significant (*P*=0.02) throughout the entire duration of the contraction at each contraction velocity (Fig. 2). The differences were insignificant for only short periods of the contraction at 210° s^−1^ (1-2%, *P*=0.002; 5%, *P*=0.004; 13-15%, *P*=0.002; 18-19%, *P*=0.002; 24-26%, *P*=0.002; 27-31%, *P*=0.002; 35%, *P*=0.002; 37-38%, *P*=0.002; 40%, *P*=0.002; 41-42%, *P*=0.002; 49-50%, *P*=0.002).

Similarly, there was a knee joint position-SOL EMG interaction for a relatively large interval relative to the angular displacement (*F*>∼10, 0-50%, *P*=0.002; 51-61%, *P*<0.002; 64-65%, *P*=0.002; 70% *P*=0.002; 78% *P*=0.002; 88-98% *P*=0.002; Fig. S2). When overall EMG activity of SOL was averaged, mean SOL amplitudes were higher with 14% at 30° s^−1^, 19% at 60° s^−1^ and 13% at 120° s^−1^ on average in knee-flexed PF relative to knee-extended PF; the difference was marginal at 180° s^−1^ and 210° s^−1^. Post hoc analyses revealed that, during the first half of the PF, SOL overall activity was significantly higher when PF was performed with flexed knee at 30° s^−1^ (0-25%, *P*=0.002; 28-30%, *P*=0.002; 32-35=, *P*=0.002; 76-78%, *P*=0.002; 82-95%, *P*=0.002) and 60° s^−1^ (0-35%, *P*=0.002; 36-39%, *P*=0.002; 39-43%, *P*=0.002; 53-54%, *P*=0.002) (Fig. 3). At faster contraction velocities, the EMG activities were statistically different for several short intervals (120° s^−1^: 3%, *P*=0.002; 4-8% *P*=0.006; 14-15%, *P*=0.002; 19-22%, *P*=0.002; 26%, *P*=0.002; 76-78%, *P*=0.002; 80%, *P*=0.004; 180° s^−1^: 0-1%, *P*=0.002; 32-33%, *P*=0.002; 210° s^−1^: 36%, *P*=0.002; 40-41%, *P*=0.002; 48%, *P*=0.002; 55-56%, *P*=0.002; 60%, *P*=0.002; 65-67%, *P*=0.002).

## DISCUSSION

The purpose of this study was to determine the effects of knee joint position and PF contraction velocity on ankle PF torque and activation amplitude of the MG and SOL in healthy young adults. As hypothesized, PF torque was lower with the knee flexed compared with the knee extended at all contraction velocities. We also observed differences in the amplitude of the MG during knee-flexed versus knee-extended PFs, while the overall MG activity decreased in knee-flexed PF. Based on the extant data in the literature, we expected increases in SOL activity in knee-flexed PFs at lower contraction velocities, assuming that this difference disappears with increasing contraction velocity. In contrast to this hypothesis, we observed elevated overall EMG activity in knee-extended PFs only at the beginning of the contraction at lower velocity contractions.

### Effects of knee position during PF on ankle torque production

Knee-flexed and knee-extended PFs alter the architectural (Avancini et al., 2015; Kawakami et al., 1998) and neurophysiological (Arampatzis et al., 2006; Avancini et al., 2015; Hahn et al., 2011; Kunugi et al., 2022; Shinohara et al., 2006) properties of the PFs. Specifically, knee-flexed PF reduces the length of the MG fascicles (Arampatzis et al., 2006; Kawakami et al., 1998). The lower MG EMG amplitude we observed suggests a decrease in MG contribution to the total ankle torque during knee-flexed versus knee-extended PF (Fig. 2) (Shinohara et al., 2006). The reduction in MG EMG amplitude in the present study was consistent with the reductions reported previously during dynamic (Farrag et al., 2022) and isometric (Arampatzis et al., 2006; Avancini et al., 2015; Hahn et al., 2011; Kunugi et al., 2022; Shinohara et al., 2006) contractions. However, in our protocol, we used a constantly increasing contraction velocity instead of the most commonly used constant velocity. This type of contraction has different angle-time characteristics (Fig. 1), and the duration of the contraction is also markedly longer than a numerically similar constant velocity contraction (i.e. 1.5 s versus 3.5 s at 30° s^−1^). Our protocol also resulted in 31% (range: 28-33%) lower peak torques in knee-flexed versus knee-extended similar to previous isometric (Avancini et al., 2015; Kunugi et al., 2022; Shinohara et al., 2006) and dynamic (Farrag et al., 2022) PFs. While our results agree with previous studies, the peak torque at each contraction velocity was 30-40% greater than the torques reported previously during constant velocity PFs (Baxter and Piazza, 2014; Farrag et al., 2022). The magnitude of difference is similar at all contraction velocities, suggesting that the knee joint affects ankle torque similarly independent of contraction velocity. With increasing contraction velocity, peak torque proportionally decreases from 178.7 N·m to 125 N·m when the knee is extended, and from 127.7 N·m to 90.06 N·m when the knee is flexed, due to the force-velocity characteristics of the muscles (Hill, 1938; Thom et al., 2007). As a result of the longer contraction time and gradually increasing contraction velocity, muscle shortening velocity is lower, enabling the muscles to generate higher forces in accordance with the force-velocity relationship (Hill, 1938), and muscle fascicles could operate for a longer time in the optimal range of fascicle length (Drazan et al., 2019) especially when the knee is extended. This could explain why the peak PF torque obtained in this study was much higher than in previous reports with similar range of motion and contraction velocities but with constant velocity PFs (Baxter and Piazza, 2014; Drazan et al., 2019).

### Effects of knee position during PF on MG muscle activity

During knee-flexed versus knee-extended PFs, PF torque was lower for nearly the entire PF range of motion regardless of the contraction velocity, which is consistent with the EMG data. MG EMG activity was 35% (range: 13-61%) lower on average in knee-flexed versus knee-extended PFs (Fig. 2), similar to studies that used isometric (Arampatzis et al., 2006; Avancini et al., 2015; Hahn et al., 2011; Kunugi et al., 2022; Shinohara et al., 2006) and concentric (Farrag et al., 2022; Kinugasa et al., 2011) PFs. The magnitude of these differences suggests a drastically reduced force generation capacity of the MG when the knee is flexed. The reduction in EMG activity in knee-flexed PF is often attributed to the altered distribution of the neural input to ankle PFs, which is related to their mechanical efficiency (Hahn et al., 2011; Kawakami et al., 1998), and it is directly influenced by the length of MG fibers. Thus, a shorter operating fascicle length in knee-flexed PF (Kawakami et al., 1998) could be suboptimal for force production, which can also be related to the lower MG activation during knee-flexed versus knee-extended PF (Avancini et al., 2015). The knee-flexed position reduced the overall EMG amplitude compared to the knee-extended PF. The difference in MG EMG amplitudes between knee-flexed and knee-extended PF was significant at each contraction velocity (Fig. 3). However, the magnitude of this difference decreased as the contraction velocity increased, likely due to altered muscle activation. (Arampatzis et al., 2006; Avancini et al., 2015; Cresswell et al., 1995). These data suggest that lower MG EMG activity reduced joint torque in knee-flexed PFs. Contraction velocity seems to alter the neural activation pattern of the MG. At the slowest contraction velocity, we observed surface electromyographs with relatively similar root-mean square (RMS) amplitudes along the whole muscle in knee-extended PFs but with significantly lower overall RMS amplitude in knee-flexed PFs. This suggests that activation of the MG muscle in knee-flexed PFs may reduce overall MG activity not just at the local level due to a general decrease in fascicle length during concentric contraction. A simulation study (Drazan et al., 2019) demonstrated that the pennation angle of the MG increases significantly over the PF range. Therefore, the results of MG muscle activation should be interpreted in the context of its pennated architecture. During concentric PFs, the distal region of the MG seems to be more active at higher versus lower contraction velocities regardless of knee joint position but with a lower overall RMS amplitude in knee-flexed PF. In the knee-flexed position, the location of the MG myotendinous junction shifts distally (Avancini et al., 2015), which would lower the tendon load and consequently increase the muscle-tendon compliance (De Monte et al., 2006; Avancini et al., 2015).

The observed decrease in PF torque with the knee flexed is often attributed to a shorter operational fascicle length of the gastrocnemius, leading to reduced force transmission to the Achilles tendon (Kawakami et al., 1998) and a concomitant decrease in neural drive to the gastrocnemius (Cresswell et al., 1995; Kennedy and Cresswell, 2001; Lauber et al., 2014). Specifically, Kennedy and Cresswell (2001) reported significantly higher motor unit recruitment thresholds for MG in the knee-extended position compared to the knee-flexed position, indicating an inhibitory effect on lower-threshold motor units during flexion. Both low and high threshold motor units appear to be inhibited during knee-flexed PF (Hali et al., 2019). This inhibition may arise from altered afferent feedback, including reduced input from muscle spindles or heteronymous reflex arcs from plantar mechanoreceptors (Shoji et al., 2005). Such feedback likely suppresses functionally inefficient muscle fibers, minimizing metabolic costs of the compromised muscle fibers (Hali et al., 2019) to maintain movement efficiency. In contrast, the SOL motor unit pool appears to be unaffected by knee joint position, as no changes in recruitment threshold or firing rate were observed (Hali et al., 2019), suggesting differential modulation of MG and SOL motor pools under flexed conditions. Reflex contributions, particularly Ib interneuron inhibition, further explain the reduced EMG recruitment in knee-flexed PF. Shoji et al. (2005) demonstrated that low-strength plantar nerve stimulation evokes short-latency inhibition in the SOL via Ib interneurons rather than Ia pathways or cutaneous afferents. This inhibition increases under unloading conditions, highlighting the role of load-dependent feedback in modulating motor unit excitability. These findings suggest a dynamic interaction between neural drive and reflex pathways in modulating task-specific muscle activation during knee-flexed PF.

### Effects of knee position during PF on SOL muscle activity

Knee position did not seem to affect the fascicle operating length of the SOL (Lauber et al., 2014). The present results cannot confirm our hypothesis that SOL EMG activity would increase during knee-flexed PF in compensation for reductions in force generation by the MG (Avancini et al., 2015; Shinohara et al., 2006). A few studies have examined SOL EMG activity patterns during dynamic PFs, but the results are inconsistent. Farrag et al. (2022) found increased SOL activity in knee-flexed versus knee-extended PFs. These data agree with previous reports on isometric contraction (Kennedy and Cresswell, 2001; Lauber et al., 2014), although others found no effects of knee position on SOL activity (Arampatzis et al., 2006; Cresswell et al., 1995; Hali et al., 2019). Interestingly, and in contrast to previous reports, we observed lower overall EMG amplitude at the beginning of the contraction in knee-extended compared to knee-flexed PF. The contraction conditions used in this study are different from previous studies in which researchers mostly utilized an isometric (Arampatzis et al., 2006; Lauber et al., 2014; Shinohara et al., 2006) or dynamic (Farrag et al., 2022) protocol. Therefore, we need to consider different muscle-tendon mechanics as force-length and force-velocity characteristics manifest differently. First, the duration of the contraction is longer, enabling the generation of muscle force for a longer time, enhancing force output, which is supported by our findings as higher ankle joint torque was achieved by the participants than in previous studies (Avancini et al., 2015; Farrag et al., 2022; Kunugi et al., 2022; Shinohara et al., 2006). In addition, we speculate that the actin-myosin interaction and the formation of cross-bridges are probably different than in an isokinetic contraction, which might also have influenced torque development. Under isokinetic contraction, muscle fascicle length continuously changes at a similar rate; but, in the protocol that we used, the rate of fascicle shortening should be gradually increasing during joint movement. However, this does not explain why we observed higher EMG activity in knee-extended PF. The difference in EMG amplitude is greatest when the ankle is in a dorsiflexed position; thus, the muscle-tendon unit is at a longer length than in a neutral joint position. Since MG is presumably at a shorter length in a knee-flexed position, we can assume that Achilles tendon stiffness is lower (Liu et al., 2020). When the knee is in a flexed position, the MG muscle-tendon junction is located more distally (Avancini et al., 2015), indicating a lower load on the tendon, thus a lower tendon stiffness, which could determine a large amount of the whole muscle tendon compliance (Farcy et al., 2014). In this case, the overall triceps surae stiffness could be lower (Hirata et al., 2016; Liu et al., 2020), with smaller muscle tension in the SOL as well (Hirata et al., 2016; Liu et al., 2020). Since the contraction starts in a dorsiflexed position, SOL fascicles are elongated; thus, the SOL probably needs to operate under a longer than the optimal length at the beginning of the contraction, which could account for the lower EMG amplitude of the SOL at the beginning of the slower knee-flexed PF. The SOL contains the highest percentage of slow-twitch fibers relative to the muscle size (Gollnick et al., 1974; Ohira et al., 1999; Tirrell et al., 2012); thus, under a given time, fewer cross-bridge connections can occur than in other muscles, and slow muscles have lower rates of time-dependent cross-bridges (Rall, 1985). This could explain that, at higher contraction velocities, when the contraction time is much shorter and the contraction is far faster, the difference in EMG activity disappears between knee-flexed and knee-extended PF. This finding is in agreement with similar observations where SOL EMG activity was unaffected by knee joint position (Arampatzis et al., 2006; Hali et al., 2019). Similarly to in the MG, SOL fascicles can also become more parallel with the surface during PF, and consequently with the electrode, which should be taken into account when interpreting our results.

Our findings, while consistent with previous reports (Avancini et al., 2015; Farrag et al., 2022), offer novel insights into the influence of knee joint position (flexed versus extended) and ankle muscle contraction velocity on PF torque. We found that using non-constant (i.e. non-isokinetic) PF contraction, the duration of PF muscle contraction increases. The longer contraction duration compared with isokinetic conditions allows the PF muscles to generate greater torques, which in turn amplifies the effects of knee joint position on PF torques. That is, under such non-constant PF contractions, PF torques generated with the knee extended versus flexed are even greater compared with the torques reported by previous studies using isokinetic testing conditions (Baxter and Piazza, 2014; Drazan et al., 2019; Farrag et al., 2022). Even after normalizing torque data to body mass, our results for both knee-flexed and knee-extended PF remain higher. For instance, when measuring peak torque of PF with the knee extended at a contraction velocity of 30° s^−1^, we obtained a value of 2.30 N·m kg^−1^, whereas other studies reported values ranging from 1.41 to 1.70 N·m kg^−1^ (Baxter and Piazza, 2014; Drazan et al., 2019). At 60° s^−1^, our measurement was 2.16 N·m kg^−1^, compared to 1.32 to 0.74 N·m kg^−1^ in other reports (Drazan et al., 2019; Farrag et al., 2022). At higher contraction velocities, we also noted increased values: 1.96 N·m kg^−1^ compared to 1.21 N·m kg^−1^at 120° s^−1^, and 1.61 N·m kg^−1^ compared to 1.00 N·m kg^−1^ at 210° s^−1^ (Baxter and Piazza, 2014). In the knee-flexed position, we observed a torque of 1.64 N·m kg^−1^, which is nearly three times higher than the 0.6 N·m kg^−1^ reported by Charles et al. (2020) at a speed of 30° s^−1^. At 60° s^−1^, our measurement was 1.45 N·m kg^−1^, compared to 1.26 N·m kg^−1^ reported by Farrag et al. (2022). We observed that the difference in PF torque between knee-flexed versus knee-extended PFs was greater at slower PF angular velocities, i.e. ∼56% at 60° s^−1^ and ∼40% at 210° s^−1^. This velocity dependence suggests that the SOL, predominantly comprising slow-twitch muscle fibers (Gollnick et al., 1974; Ohira et al., 1999; Tirrell et al., 2012), compared with the MG might be more affected by contraction velocity. Interestingly, it is often suggested to perform PF strength training with the knee extended in younger adults (Signorile et al., 2002); such configuration of the lower extremity may not be optimal in older adults. Ema et al. (2020) reported that MG and LG EMG activity seems to be higher in knee-flexed versus knee-extended PFs in older versus younger adults. These data should be confirmed during dynamic conditions as well. It seems that we need more information to determine if PF contraction duration and velocity interact with age, sex, and training history to optimize PF strength training.

### Limitations

One limitation is that while the axis of rotation of the motor was aligned as coaxially as possible with the axis of rotation of the right ankle, there is still some deviation from the true axis of rotation of the ankle joint, which could change as the ankle rotates. However, we think that these errors were consistent during the measurements and would thus have minimal effects on the conclusions. We measured EMG activity with surface array electrodes. The skin-to-muscle displacement and fascicle orientation influence the EMG signal during isometric PF (Avancini et al., 2015), and it is possible that, during dynamic contraction, changes in the electrode positions in relation to the active muscle fascicles are greater. Therefore, the reduced EMG amplitudes in the EMG activity during flexed-knee PF could be affected by the number of recorded motor units. However, we note that the reductions in MG EMG were similar to those reported previously (Avancini et al., 2015; Kunugi et al., 2022; Shinohara et al., 2006). To minimize the effects of muscle shift under the skin on EMG activity, we used the average activity of channels 1-15. Visualizing and analyzing the fascicle behavior of the MG and SOL could provide details about the orientation of the fascicle to observe the magnitude of the skin to muscle displacement and would help to explore the force-length characteristics of these muscles, which could improve understanding and interpreting our results. Other knee joint positions would influence MG and SOL EMG activity patterns differently (Farrag et al., 2022); thus, the results of this study cannot be generalized to different knee joint positions. We acknowledge that the use of isokinetic dynamometers, while ensuring high internal validity and precise control of movement parameters, inherently limits the external validity of our findings. The constrained and unique nature of the testing environment does not approximate the complex, dynamic conditions of real-life movements, limiting the broader applicability of our results.

## Conclusions

We conclude that knee position affects MG and SOL activation during dynamic PF, with PF torque decreased in the knee-flexed position. This reduction in torque can be attributed to the lower EMG activity of the MG. However, we found no evidence that changes in SOL activation would compensate for the decrease in MG activation.

## MATERIALS AND METHODS

### Participants

Healthy male volunteers participated in this study (*n*=30, 29.1±1 years of age, 182.4±7.9 cm height, 77.8±9.4 kg body mass). They engaged in recreational physical activity three or more times per week. Participants were recruited from the university student population through voluntary sign-up. They were free of pain and had no injuries to the lower extremities over the past 2 years. Exclusion criteria included having any previous lower limb muscle injuries within the past 2 years or currently experiencing pain or potential injury in these muscles. Prior to the experiment, participants received verbal and written explanation about the experimental procedures. Each participant signed an informed consent document in line with the Declaration of Helsinki. This study was approved by the local ethics committee of the Hungarian University of Sports Science (TE-KEB/13/2022). *A priori* sample size calculations (G*Power 3.1.7) (Faul et al., 2009) revealed that a minimum sample size of 28 participants would be needed to detect significant differences in MG and SOL activity, assuming a moderate effect size (0.55), type I error of 0.05, and power of 0.80.

### Study design

**Fig. 4.**
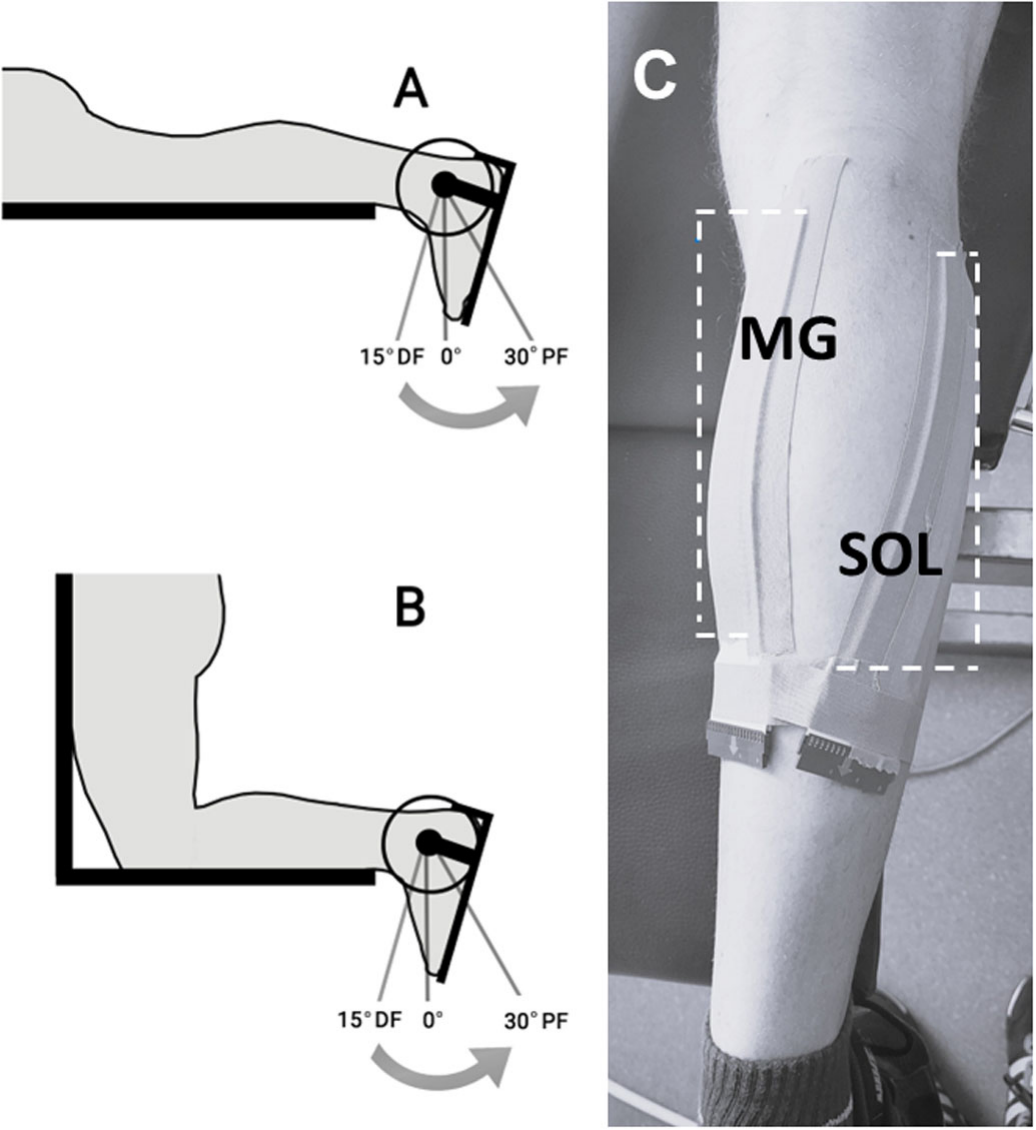
**Knee joint position and electrode placement.** (A) Knee-extended plantarflexion with a range of motion from 15° dorsiflexion (DF) to 30° plantarflexion (PF). (B) The same range of motion but in the knee-flexed position. (C) Electrode placement over the medial gastrocnemius (MG) and soleus (SOL) muscles for the HD-EMG recordings.

Participants visited the biomechanics laboratory once. Prior to testing, participants cycled on a stationary bike for 5 min (80 W, ∼60 rpm) and stretched for 5 min. For the experimental task, they performed concentric PF with the knee flexed or extended on a custom-built computer controlled isokinetic dynamometer (Multicont II dynamometer, Mediagnost, Mechatronic Kft., Szeged, Hungary). The specific details of the dynamometer, including its technical and operational characteristics, as well as the calibration procedures, were reported previously (Rácz et al., 2002). In a prone position, the right foot was strapped to a custom-built ankle adapter, attached to the lever arm of the servo motor. The ankle was strapped to prevent the heel and toes from lifting off the adapter during PF. A built-in laser beam guided the alignment of the axis of rotation of the servo motor with the axis of rotation of the right ankle. To prevent upward movement on the bench and to ensure that force generation relied solely on the calf muscles, participants were secured by straps to the dynamometer frame. Anatomical zero was set to 0° ankle angle (90° between foot and shank). Participants then practiced the task by performing three submaximal 30-80% of their perceived maximal isometric force and three concentric PFs at 30° s^−1^. Two knee joint positions were used to vary MG muscle length (Fig. 4). The contractions were completed in two randomly ordered protocols (A,B). Protocols A and B consisted of concentric contraction with extended knee (A) or flexed knee (B). Each participant completed two trials for each contraction, with a minimum inter-trial rest of 2 min (or longer, if necessary). Additionally, a minimum of 10 min was provided between protocols A and B while the dynamometer was set for subsequent PF condition.

### Dynamometric measurement

**Fig. 5.**
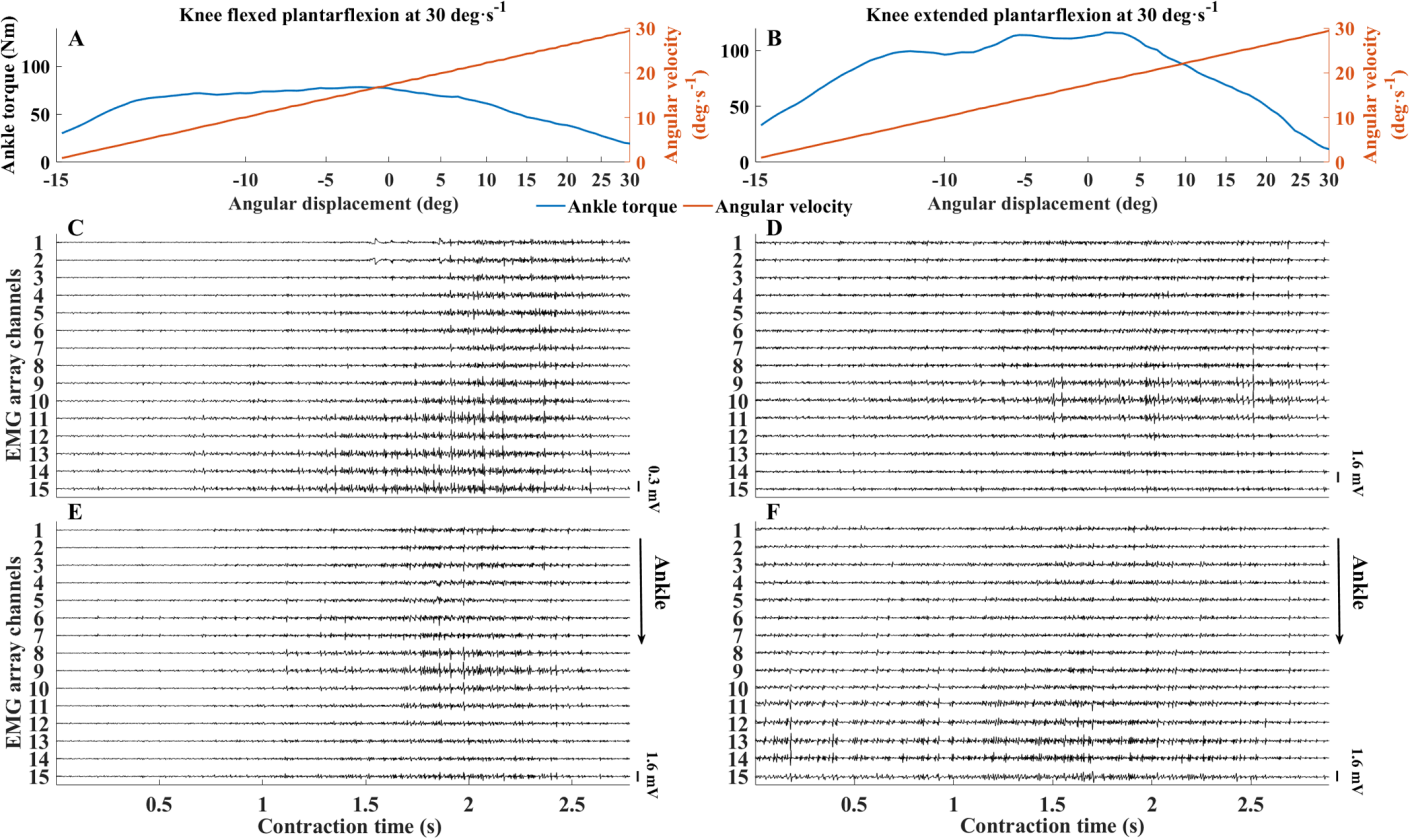
**Changes in torque and surface electromyographs with changes in knee position.** (A-F) Raw torque (A,B) and surface electromyographs (C-F) for one individual are shown during plantarflexion contractions exerted with the knee flexed at 90° (A,C,E) and knee fully extended (B,D,F). Torque and angular displacement are shown relative to angular displacement, while electromyographs are shown relative to contraction time. Panels C and D show the EMG activity of the MG at each channel; panels E and F show the EMG activity of the SOL.

Each contraction started at 15° dorsiflexion and stopped at 30° PF. Instead of a constant contraction velocity, we used continuously accelerating contraction velocities to simulate a more realistic-like muscle contraction compared to a constant velocity contraction. Each participant performed five trials each at the five velocities: 30, 60, 120, 180, and 210° s^−1^ (Fig. 5). Compared to a constant velocity contraction, in the accelerating contraction velocity testing mode, contraction durations increase. The dynamometer was programmed for participants to reach the target peak velocity by the end of the range of motion so that the velocity continuously increased from the start of the contraction (Fig. 5). The lever arm started to move after participants reached the 30 N·m torque threshold. PFs with the knee extended and flexed were executed in a prone and a kneeling position, respectively (Fig. 4). In each knee position and contraction velocity, participants performed three PF trials at a maximal effort with verbal encouragement. Torque angle data were recorded at a sampling frequency of 1000 Hz. From the torque-angle data peak torque and mechanical work (area under the torque-angle curve) during each contraction was calculated using a custom script in MATLAB (v2022a, MathWorks, Natick, MA, USA). The raw data from the dynamometer were filtered using a zero-delay fourth-order Butterworth filter with a cut-off frequency of 10 Hz.

### Electromyography

We recorded surface EMG activity from the MG and SOL with a portable HD-EMG system (sessantaquattro+, OT Bioelectronica, Torino, Italy). A 16-channel linear EMG array (16 silver-bar electrodes, 10×1 mm, 10 mm inter-electrode distance; Spes Medica, Battipaglia, Italy) was mounted on each muscle with double-sided tape (OT Bioelectronica) positioned parallel to each muscle's longitudinal axis. Conductive paste (TEN 20 Conductive Paste, Weaver, Italy) ensured the electrical contact between electrodes and the skin. Before electrode placement, the skin was shaved, abraded lightly and cleaned with alcohol to minimize skin impedance. The reference electrode was placed over the right medial malleolus of the contralateral limb. We affixed the electrode arrays using ultrasonography (EUB 405 plus, Hitachi-Aloka, Tokyo, Japan) to identify and mark the borders of the MG and SOL muscles and attach the arrays as far from the muscle borders as possible to minimize cross talk from neighboring muscles. For the MG, the most proximal electrode was positioned as proximally as possible near the femoral condyle, to avoid array folding during knee flexion. For the SOL, the most proximal electrode was positioned as proximally as possible near the fibula head and following the superficial region of the muscle. Because the proximal part of the SOL is potentially subjected to cross talk from the neighboring peroneus muscle, the signal to noise ratio was checked before the testing procedures. Some participants had a smaller area of superficial SOL and/or MG than the electrode length, which was inspected via ultrasound; thus, the overlapped channels on the muscle were marked and excluded from data analysis. The EMG arrays were connected to a 12-bit A/D converter (OT Bioelettronica), and the signals were amplified (×1000) then recorded in OT BioLab+ software (v1.5.6.0, OT Bioelectronica). During the measurements, 15 differential channels of EMG signals were recorded from each muscle (sampling frequency: 2 kHz). The torque and angle analog signals provided by the dynamometer machine were sampled synchronously with the electromyographs using an isolated adapter (ISO-AUXSP, OT Bioelettronica). All signals were inspected prior to acquisition and corrected for contact problems and power line interference. The collected raw HD-EMG signals were band-pass filtered (10-500 Hz) using a zero-phase fourth-order Butterworth filter in MATLAB. The RMS amplitude of the EMG signals was calculated for each channel. Since there is a relevant difference in contraction durations, using a single RMS window size could bias the results towards longer duration contractions. To mitigate this, we proportionately reduced the RMS window size for shorter duration contractions. For the slowest contraction (30° s^−1^, a 100 ms RMS window was applied, and then the RMS window was reduced proportionately according to the duration of the contraction at a higher contraction velocity (for example, 50 ms for 60° s^−1^). Overall EMG activity of the MG and SOL was defined for each muscle as the average normalized RMS activity of the 15 channels. Overall EMG activity was used to calculate differences between PF conditions because of the relatively large muscle shift under the skin, which could affect the regional EMG activity. Torque and EMG curves were time normalized (0-101 points) for the contraction duration, but the results are shown relative to ankle angular displacement for better interpretation (the angle data was proportionally calculated to the contraction cycle 0-100%).

### Statistical analyses

After checking the normal distribution of the data, all statistical analyses were performed using Statistical Parametric Mapping (SPM 30, v.0.4.8; www.spm1d.org) in MATLAB to compare time-normalized torque and EMG curves. Because most data were not normally distributed, we used the non-parametric version of the SPM tests (SnPM). Two-way repeated-measures ANOVA were run across the PF conditions. SnPM{F} test statistics were calculated to test knee joint position muscle-specific EMG interactions and velocity-muscle-specific EMG interactions (for both muscle). Family-wise type I error rate was set at 0.05. In all SnPM analyses, the test statistic (SnPM{F} or SnPM{t}) was first computed, and the critical threshold (F* or t*) was determined. In case of an interaction at any timepoint across the PF, locations of the differences were tested using paired-sample SnPM{t} test with Bonferroni correction (*P*=0.01). Whenever the test statistic trajectory crossed the critical threshold (forming so-called suprathreshold clusters), the difference was considered statistically significant. Finally, *P*-values were calculated for each suprathreshold cluster. SPM technical details are described elsewhere (Pataky et al., 2016a,b).

## Supplementary Material

10.1242/biolopen.061810_sup1 Supplementary information
