# Supplementary material for: Knee position affects medial gastrocnemius and soleus activation during dynamic plantarflexion: no evidence for an inter-muscle compensation in healthy young adults
Source: Biol Open. 2024 Dec 30;13(12):BIO061810. doi: 10.1242/bio.061810 (PMC11708772; doi:10.1242/bio.061810)
Supplement: Supplementary information [file biolopen-13-061810-s1.pdf]

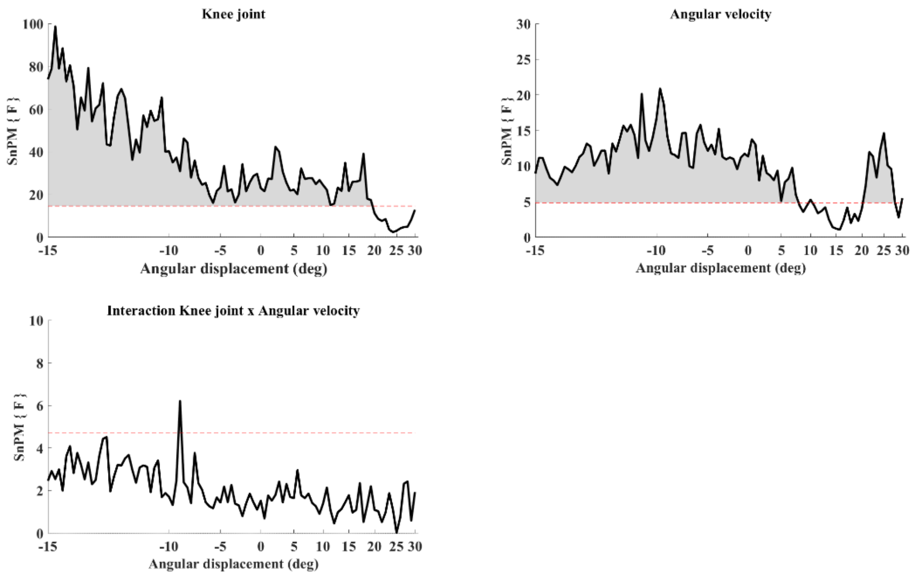

Fig. S1. Result of the ANOVA test for medial gastrocnemius. Thick black lines are the SPM{F} test statistics representing the magnitude of the effects relative to the SD and sample size (n = 30). Critical thresholds (F*) were calculated (dashed red horizontal lines; family-wise α = 0.05). Probability (p) values are shown for each suprathreshold cluster depicting statistically significant effect. There is a significant knee joint effect on MG EMG activity from 4-100% of the contraction. Angular velocity has a significant effect on MG EMG activity from 0 to 72% at 77%, 89 to 98% and from 99 to 100% of the contraction. A knee joint x angular velocity interaction was only seen in a brief period at 35% of the contraction. Spacing between the x ticks on each panel are gradually decreasing toward the end of the contraction representing the continuously accelerating contraction velocity.

Biology Open • Supplementary information

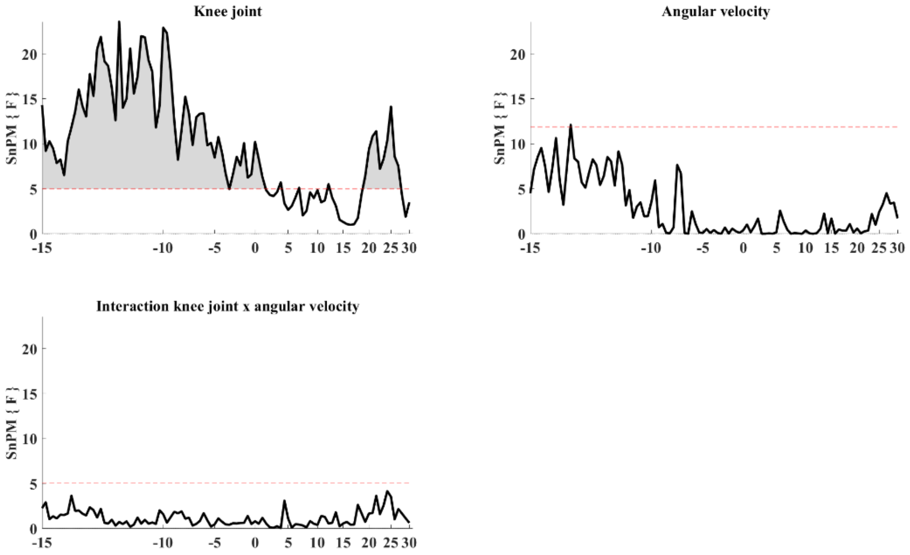

Fig. S2. Result of the ANOVA test for soleus. Thick black lines are the SPM{F} test statistics representing the magnitude of the effects relative to the SD and sample size (n = 30). Critical thresholds (F*) were calculated (dashed red horizontal lines; family-wise α = 0.05). Probability (p) values are shown for each suprathreshold cluster depicting statistically significant effect. There is a significant knee joint effect on MG EMG activity from 0 to 50%, 51-61%, 65%, 70%, 78% and from 88 to 98% of the contraction. Angular velocity has a significant effect on MG EMG activity only for a brief instant at 11% of the contraction. No knee joint x angular velocity interaction was detected. Spacing between the x ticks on each panel are gradually decreasing toward the end of the contraction representing the continuously accelerating contraction velocity.

Biology Open • Supplementary information
